# The British Medical Association

**Published:** 1908-08-01

**Authors:** 


### The British Medical Association Meeting.

The presidential address delivered at Sheffield on July 29 by Mr. Simeon Snell, who is also the Middlemore prizeman of the year, dealt with certain important investigations which he has carried out as to the vision of coalminers. It would seem that "deputies"—that is, those whose duty it is to discover and report on the presence of firedamp in the working galleries—have occasionally been fined for neglect of duty when in reality their omission to notice the gas has been due to visual defect, not negligence. Mr. Snell has, with the assistance of Mr. A. H. Stokes, an Inspector of Mines, examined a large number of colliers, and he finds a peculiar nystagmus prevailing among them. When this condition exists, the vision of stationary objects is so affected that they appear to oscillate, and in consequence the "gas-cap" on the safety-lamp may pass undetected even when it exists in a marked degree. The cause of this nystagmus is supposed to be weariness of the elevator muscles of the eye set up by the constrained position in which miners work. Experimental research was conducted at the Sheffield Royal Infirmary, and the vision of forty-eight colliers from thirteen collieries was tested. It was found that their incapacity to detect the "gas-cap," unless a dangerous quantity of firedamp was present, was very marked in all of them. As Mr. Snell pointed out, it is difficult to judge how far explosions have been due to this defect of vision, because in such cases the "deputy" generally loses his life with the other victims. He suggested the need for periodical medical inspection of mine officials in emphatic terms, and it affords us some legitimate satisfaction to find that the President of the B.M.A. shares a view which we have already put forward in The Hospital (June 20, 1908, p. 299). The address in medicine by Dr. Kingston Fowler was, as might be expected, a thoughtful and scholarly review of current medical progress; especially in relation to tuberculosis, and its treatment by tuberculin controlled by opsonic determination. Dr. Fowler is a physician who combines a thoroughly scientific spirit of scepticism with a willingness to examine every new departure on its merits, and it is interesting to note that he is very decidedly favourable to the tuberculin treatment as now carried out, whereas a decade or so since he severely condemned the original form of it as useless and harmful. Passing from medicine pure and simple to questions of the future of the medical profession, Dr. Fowler hinted at a modification of existing customs which has been suggested before—namely, the combination of medical and surgical practice on a given region as the true province of the specialist, instead of the present system by which a consultant is restricted to one branch of practice, but may apply it to any part of the body. He pointed out that in gynæcology this process has already quietly been accomplished. On Prophylaxis in Surgery Mr. R. J. Pye Smith delivered a well-reasoned address, which was remarkable for the wide range the orator was able to cover. Many of the topics which have lately absorbed attention in the past year, such as ophthalmia neonatorum, spinal anæsthesia, and so on, were passed under attention in succession. Especially noteworthy was his exhortation to surgeons to realise how terrifying the prospect of an operation frequently is to the laity, and how important it is to encourage the patient and pacify the fears of those who are about to undergo this ordeal.

